# Combined analysis of transcriptomics and metabolomics on the cumulative effect of nano-titanium dioxide on mulberry seedlings

**DOI:** 10.3389/fpls.2023.1175012

**Published:** 2023-06-14

**Authors:** Dongliang Yu, Qingyu Lu, Yuting Wei, Di Hou, Xingcan Yin, Kunpei Cai, Changyu Qiu, Kaizun Xu

**Affiliations:** ^1^ College of Agriculture, Guangxi University, Nanning, Guangxi, China; ^2^ Sericulture Technology Promotion Station of Guangxi Zhuang Autonomous Region, Nanning, Guangxi, China; ^3^ Guangxi Key Laboratory of Agro-Environment and Agric-Products Safety, National Demonstration Center for Experimental Plant Science Education, College of Agriculture, Guangxi University, Nanning, Guangxi, China

**Keywords:** TiO_2_ NPs, mulberry, seedling growth, transcriptomics, metabolomics

## Abstract

**Introduction:**

Titanium dioxide nanoparticles (TiO_2_ NPs) are among the most widely used inorganic nanomaterials in industry, medicine and food additives. There are increasing concerns regarding their potential risks to plants and the environment. Mulberry trees are widely grown in China due to their high survival rate and ability to aid ecological recovery.

**Methods:**

Herein, the effects of TiO_2_ NPs with different concentrations (100, 200, 400 and 800 mg/L) on the growth and physiology of the mulberry tree were systematically evaluated in aspects of physiology, transcriptomics and metabolomics.

**Results:**

Results showed that TiO_2_ NPs could be absorbed by the mulberry sapling root system and be transferred to the plant shoot. This results in the destruction of mulberry sapling root and leaf tissue. Furthermore, the number of chloroplasts and their pigment contents were reduced and the homeostasis of metal ions was disrupted. The toxic effects of TiO_2_ NPs attenuated the mulberry sapling’s stress resistance, the contents of malondialdehyde in 100 mg/L, 200 mg/L 400 mg/L and 800 mg/L treatment groups increased by 87.70%, 91.36%, 96.57% and 192.19% respectively compared with the control group. The transcriptomic data showed that TiO_2_ NPs treatment mainly affected the expression of genes related to energy synthesis and transport, protein metabolism, and response to stress. Meanwhile, the results of metabolomics showed that 42 metabolites produced significant differences in mulberry, of which 26 differential metabolites were up-regulated in expression and 16 differential metabolites were down-regulated, mainly including metabolic pathways such as secondary metabolite biosynthesis, citric acid cycle, and tricarboxylic acid cycle, and was not conducive to the seed germination and or growth of the mulberry sapling.

**Discussion:**

This study enriches the understanding of the effects of TiO_2_ NPs on plants and provides a reference for the comprehensive scientific assessment of the potential risks of nanomaterials on plants.

## 1 Introduction

Nanoparticles (NPs), along with genetic engineering and artificial intelligence, are commonly referred to as the cutting-edge technologies of the 21st century. NPs have the properties of stability, hydrophilicity, photocatalysis, nonirritant, good weatherability and shading in mechanics, heat, magnetism, optics, electricity and chemistry (Huang et al., 2019; Baharlooeian and Badhul, 2020). They can be applied in targeted drug delivery, diagnosis, tissue engineering and environmental remediation (Gandhimathi et al., 2019; Bidast et al., 2020; Santelli et al., 2020; Bajpai et al., 2022; Shukla et al., 2022).

Titanium dioxide nanoparticles (TiO_2_ NPs), represent one of the most common nanoparticle types (Shi et al., 2016). They mainly exist in anatase, rutile and brookite crystal forms. Among these, anatase TiO_2_ has the highest nanoparticle energy gap and catalytic activity (Kovačič et al., 2022). TiO_2_ NPs are currently widely used and the global output is increasing year by year. TiO_2_ NPs have anti-UV, antibacterial, self-cleaning, anti-aging, anti-fading, chemical inertness, photocatalytic activity and other properties. They can be used in sunscreen, paint, fine ceramics, antibacterial fiber, plastic, paper, food additives and pollutant treatment (Lan et al., 2013; Wang et al., 2016; Torres et al., 2019; Kovačič et al., 2022). This multitude of uses results in TiO_2_ NPs possessing high economic value. However, with the wide application of TiO_2_ NPs, their safety profile has attracted greater attention recently. It has been reported that TiO_2_ NPs may be more toxic to organisms than TiO_2_ coarse particles (Wei et al., 2018; Baharlooeian and Badhul, 2020). Its safety has been widely studied in agriculture, medicine, environmental and other fields (Dreno et al., 2019; Fattahi et al., 2021; Fiordaliso et al., 2022), and is a global current research hotspot (Wu et al., 2014; Shi et al., 2022). TiO_2_ NPs can enter soil, water and the atmosphere through sewage treatment, soil pollution treatment, waste landfill and the use of related products (Shi et al., 2016). Due to TiO_2_ NPs being difficult to degrade, they can become enriched in the environment with concentrations reaching hundreds of milligrams per kilogram (Piccinno et al., 2012; Keller and Lazareva, 2014; Wang et al., 2014; Zhou et al., 2015; Aragaw et al., 2021; Nabi et al., 2021). By posing a threat to the environment, it is an emerging environmental pollutant (Frazier et al., 2014).

There are controversies regarding the effects of TiO_2_ NPs on plants that are influenced by many factors (Song et al., 2013; Bueno et al., 2022), such as plant species and TiO_2_ NPs concentration (Nowac, 2009). For example, 5 mg/L of TiO_2_ NPs significantly inhibited the growth of algae, the depletion of antioxidant enzymes with a concomitant increase in malondialdehyde levels and reactive oxygen species posed a hazard to membrane integrity (Xia et al., 2015), and 100-1000 mg/L of TiO_2_ NPs reduced the growth and chlorophyll content of *Arabidopsis thaliana*, while 1000 mg/L TiO_2_ NPs was beneficial to the growth of its roots and affected vitamin E content by the regulation of the expression level of its biosynthetic genes (Szymańska et al., 2016). Meanwhile, 5-2500 mg/L TiO_2_ NPs was demonstrated to promote photosynthesis in spinach, tomato and lettuce (Hong et al., 2005; Yang et al., 2006; Qi et al., 2013; Pan et al., 2020), however, 100 mg/L TiO_2_ NPs treatment enhanced photosynthesis of tomatoes, while 200 mg/L TiO_2_ NPs treatment inhibited photosynthesis of tomatoes (Choi, 2021). Moreover, 25 µg/L TiO_2_ NPs promoted seed germination and biomass increase, but 200 µg/L TiO_2_ NPs increased antioxidant enzyme activity, led to lipid peroxidation and DNA damage (Tiwari et al., 2017), and 1000 mg/L TiO_2_ NPs was exposed to grape leaves, the nanoparticles reduced the electron transfer rate and photosynthetic efficiency of chloroplasts (Teszlák et al., 2018). Interestingly, 0-5000 mg/L TiO_2_ NPs not only showed no toxicity to lettuce, rape, kidney bean and rice, but also enhanced the photosynthesis efficiency of rice (Song et al., 2013; Xu et al., 2019).

Mulberry (*Morus Alba* L.) belongs to the Mulberry genus of Moraceae, which is distributed in most tropical and subtropical regions of the world. It is the most widely planted shrub and also is an important cash crop in China, covering more than 626,000 ha, and has been planted in China for over 4,000 years (Yuan et al., 2015; Liu et al., 2017; Pel et al., 2017). Its leaves can not only be used as fodder for silkworm, but also used as medicine for its hypoglycemic, hypolipidemic and antioxidant effects. And the bark can be used as medicine, the trunk can be used for papermaking, and mulberry fruit can be directly used for fermentation and brewing (Arabshahi-D et al., 2007; Liu et al., 2017). Mulberry trees have a high survival rate and strong stress resistance. They can withstand harsh environmental conditions such as drought, flooding and high salt, and have a strong ability to preserve soil and play a role in ecological restoration (Guha et al., 2010; Yang et al., 2014; Li et al., 2018). And also can be used for reservoir shelterbelt forest construction (Wang et al., 2012a; Jiang et al., 2015). It is worth noting that TiO_2_ NPs have shown great potential in agriculture (Li et al., 2016; Wu et al., 2017), but no studies reporting the effect of TiO_2_ NPs accumulation on mulberry have been performed.

Seed germination is the first stage of the plant growth cycle and one of the most sensitive periods to external factors (Shuai et al., 2016). TiO_2_ NPs is the most produced and widely used nanomaterial, but its environmental impact and mechanism of action are not yet clear. In the present study, mulberry seeds were treated with different concentrations of TiO_2_ NPs suspension, and mulberry was used as the model plant to study its effect on mulberry seed germination and growth and development process, and transcriptomics and metabolomics were used to elucidate the toxicological mechanism of TiO_2_ NPs on mulberry. This study enriches the toxicological understanding of the effects of TiO_2_ NPs on the ecological environment and provides a reference for the comprehensive scientific assessment of the potential risks of nanomaterials on the ecological environment.

## 2 Materials and methods

### 2.1 Materials and reagents

TiO_2_ NPs used in this study was anatase type, which was purchased from Hangzhou Wanjing New Material Co., Ltd. (Hangzhou, China) with a purity of 99.9%. Mulberry seeds were provided by Sericulture Technology Promotion Station of Guangxi, and the variety is Guisang No.5. Hoagland culture medium was purchased from Qingdao Hope Bio-Technology Co., Ltd (Qingdao, China). Hydrogen peroxide (30%), calcium carbonate and calcium nitrate were purchased from Chengdu Jinshan Chemical Reagent Co., Ltd. (Chengdu, China). The catalase (CAT) kit, malondialdehyde (MDA) kit, proline (Pro) kit and soluble protein (Cpr) kit were purchased from Suzhou Grise Biotechnology Co., Ltd. (Suzhou, China).

### 2.2 Characterization of TiO_2_ NPs

The phase analysis of TiO_2_ NPs was examined by X-ray diffraction (XRD) (SmartLab3, Rigaku, Tokyo). With the target of Cu, a tube current of 80 mA, a scanning range of 10°~90° and a scanning speed of 10°/min. The agglomeration potential of TiO_2_ NPs in culture media, including their hydrodynamic size, polydispersity index and Zeta potential, was measured by dynamic light scattering (DLS) (ZS90, Malvern, Germany). A small quantity of TiO_2_ NPs was diluted with ultrapure water until fully resuspended. After 30 min of ultrasound, the suspension was dropped carefully into the scanning electron microscope (SEM) sample tray with conductive adhesive, smeared evenly and dried in an oven at 55°C. The dried sample was placed next into a vacuum evaporator, before being sprayed with a layer gold film of 100 angstroms. Characterization was performed using site emission scanning electron microscopy (FEI Quattro S, Thermo Fisher, Czech Republic).

### 2.3 Mulberry seed treatment

TiO_2_ NPs master stock suspension of 1000 mg/L was prepared and then diluted to 100 mg/L, 200 mg/L, 400 mg/L and 800 mg/L working suspension with ultra-pure water after ultrasound for 30 min at 120 W and 10 s in 60 s intervals.

1500 fully grained mulberry seeds were selected and soaked in 3% hydrogen peroxide solution for 3 h to disinfect the seeds prior to washing with ultrapure water 5 times. The sterilized seeds were transferred to triangular bottles containing the target suspension, with 100 seeds in each group (ultra-pure water as the control group) and three replicates for each group. The suspension was then exposed to 30°C and 150 r·min^-1^ condition on a high-speed shaking table for shock seed soaking. After 24 h the seeds were removed and their surface was cleaned, they were next placed into two 9 mm layers of filter paper in a petri dish, with the seed radicle and growth direction maintained in a straight line. Each petri dish was bathed in 4 mL ultrapure water and the dish was covered. The seeds were then placed in a constant temperature and humidity light incubator for germination. The incubator was set to a temperature of 30°C, 12 h 20% illumination, 12 h darkness, and 70% humidity. The number of seeds that had germinated was recorded every 24 h. The seeds were considered to properly germinated when the germ length was greater than 1 mm, and the culture was terminated when the number of seeds germinating ceased to increase for two consecutive days.

### 2.4 Mulberry seedling growth index measurement

Seedlings were randomly selected from the treatment group and the control group for further culture, with 3 replicates for each group and 40 plants in each replicate. The seedlings were transferred to Hoagland nutrient solution with TiO_2_ NPs at concentration of 100 mg/L, 200 mg/L, 400 mg/L and 800 mg/L, respectively. And the control group was replaced with ultra-pure water. After continued culture for 7 days, the root length, stem length and fresh weight of seedlings were measured. The germination rate, germination potential, germination index and vitality index of mulberry seedlings were calculated respectively. The calculation formulas were shown in the supplementary information of Text S1.

### 2.5 Determination of metal elements in mulberry seedlings

10 mulberry seedlings were randomly selected from each group after 14 days of culture. After washing with ddH_2_O, they were divided into shoot and root parts, placed in 2 mL centrifuge tubes and dried at 80°C before their weight was recorded. The mulberry seedlings were digested by a damp-heat digestion method, and the samples in the centrifuge tube were moved to a desiccating tube before 15 mL of mixed acid [perchloric acid (1) + nitric acid (9)] was added to the desiccating tube. After the solution was transparent and clarified, the samples continued to be heated until there remained 2-3 mL of liquid in the tube. This solution was then diluted to 10 mL with ddH_2_O. The concentrations of Ti, Fe, Mn, Cu and Zn were determined by ICP-5000 (Focused Photonics, China), and the concentration of TiO_2_ is converted from the atomic mass of Ti.

### 2.6 Stress resistance test

After 14 days culture, 5 seedlings were randomly selected from each group, and 1 mL homogenate buffer solution (0.01 mol/L Tris HCl, 0.001 mol/L EDTA-2Na, 0.01 mol/L sucrose, pH = 7.4) was added at 4°C to for homogenization in an ice bath. After centrifugation at 4°C × 12 000 rpm for 10 min, the supernatant was retrieved and the contents of catalase (CAT), proline (Pro), malondialdehyde (MDA) and soluble protein (Cpr) in supernatant were determined on ice according to kit method.

### 2.7 Determination of photosynthetic pigment content

For pigment content analysis, 0.2 g of fresh leaves from each group were placed into a mortar, 1 g quartz sand and 2 mL 95% ethanol were added prior to homogenization on ice until the tissues turned white. Next, samples were centrifuged at 10 000 rpm for 5 min, before being left to stand for 10 min. The absorbance values at 665, 649 and 470 nm, respectively, were determined for the resultant supernatants by UV spectrophotometer (UV-1800, Shimadzu, Japan), and the concentrations of chlorophyll A, chlorophyll B and carotenoid were calculated. The calculation formulas were shown in the supplementary information under Text S2.

### 2.8 Tissue section and transmission electron microscopy

Based on the results of this study, a concentration of 400 mg/L of TiO_2_ NPs was selected for follow-up experimentation. After 14 days of culture, the root and leaf tissues of fresh mulberry seedlings were taken and fixed with FAA fixative solution for 24 h. The tissues were flattened with scalpels, dehydrated with gradient alcohol, dipped in wax, and embedded before being sectioned with a pathological slicer (RM2016, Leica, Germany). The slices were stained and observed under a light microscope (DM4000, Leica, Germany). The TEM detection method was as follows: Fresh mulberry leaves cultured for 14 days were immediately placed into a petri dish with 2.5% glutaraldehyde fixative solution, cut into 1 mm^3^ tissue pieces with a scalpel, transferred to a 1.5 mL microcentrifuge tube with 2.5% glutaraldehyde fixative solution for further fixation, and temporarily stored in refrigerator at 4°C. After dehydration at room temperature, osmotic embedding, polymerization, sectioning and staining, a transmission electron microscope (HT7880, Hitachi, Japan) was used for observation and image analysis.

### 2.9 Transcriptome and metabolome sequencing

Fresh mulberry seedlings cultured in the concentration of 400 mg/L for 14 days were commissioned to be sequenced by Shenzhen BGI Technology Co., Ltd. (Shenzhen, China) for transcriptome and metabolome sequencing, with three replicates for each group. Previously described methods were used for data analysis (Li et al., 2022). The software for KEGG pathway analysis and GO analysis is ClusterProfiler.

### 2.10 qRT-PCR analysis

Among the differentially expressed genes, five differentially expressed genes, *PNC1*, *XylA*, *ABCC8*, *CcnD3* and *Hsp83*, were randomly selected for qRT-PCR verification. Trizol Kit (TaKaRa, Dalian, China) was used to extract total RNA, and PrimeScript™ RT Reagent Kit with gDNA Eraser (TaKaRa, Dalian, China) was used for reverse transcription to obtain cDNA. qRT-PCR reaction was performed in fluorescence quantitative PCR (480 ii, Roche, Switzerland). And the ChamQ Universal SYBR qPCR Master Mix was used according to the manufacturer’s instructions of Vazyme Biotech Co., Ltd. (Nanjing China). Reaction system: 10 µL 2 × ChamQ Universal SYBR qPCR Master Mix, 2 µL cDNA solution, 0.4 µL forward primer (10 µM), 0.4 µL reverse primer (10 µM), 7.2 µL ddH_2_O (Distillation-Distillation H_2_O). Reaction steps as follows: an initial denaturation step at 95°C for 30 s. The amplifications were followed by 40 cycles of 95°C for 10 s, 60°C for 30 s. And melting analysis was performed at 95°C for 15 s, 60°C for 60 s, and 95°C for 15 s. *β-actin* was used as an internal reference gene. Genes specific primers were shown in the supplementary information of **Table S1**.

### 2.11 Statistical analysis

Statistical analysis was performed using IBM SPSS Statistics 23, and the values were expressed as mean ± standard deviation (SD). One-way ANOVA was used to compare the differences between groups. * indicated that the difference reached a significant level (P < 0.05), ** indicated that the difference reached an extremely significant level (P < 0.01), which was statistically significant.

## 3 Results

### 3.1 Characterization of TiO_2_ NPs

**Figure 1:**
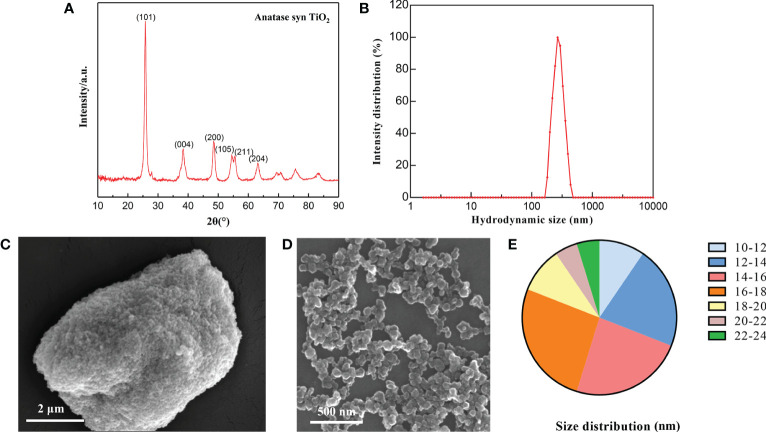
Characterization of TiO_2_ NPs. **(A)** XRD determination of TiO_2_ NPs. **(B)** Hydrodynamics determination of TiO_2_ NPs. **(C)** TiO_2_ NPs in concentrated state. **(D)** TiO_2_ NPs in dispersed state. **(E)** Particle size distribution of TiO_2_ NPs.

Firstly, the TiO_2_ NPs was characterized. XRD results revealed that the TiO_2_ NPs samples used in this study were composed of a single anatase phase with no other spurious peaks, corresponding to (101), (004), (200), (211) and (204) crystallographic planes at 2θ = 25.28°, 37.80°, 48.05°, 53.89°, 55.06° and 62.69°, respectively, and (200), (105), (211) and (204) crystal planes (**Figure 1A**), which correspond to No.21-1272 standard diffraction peaks in the JCPDS card. The hydrodynamic diameter of TiO_2_ NPs was about 293 nm, indicating the agglomeration of TiO_2_ NPs in suspension (**Figure 1B**). Subsequently, we observed the characteristics of TiO_2_ NPs by SEM. It was identified that TiO_2_ NPs mainly existed in two states of aggregation and dispersion (**Figures 1C, D**), and their size was distributed between 10-24 nm, mainly concentrated between 12-18 nm (**Figure 1E**).

### 3.2 Effect of TiO_2_ NPs on seed germination

**Figure 2:**
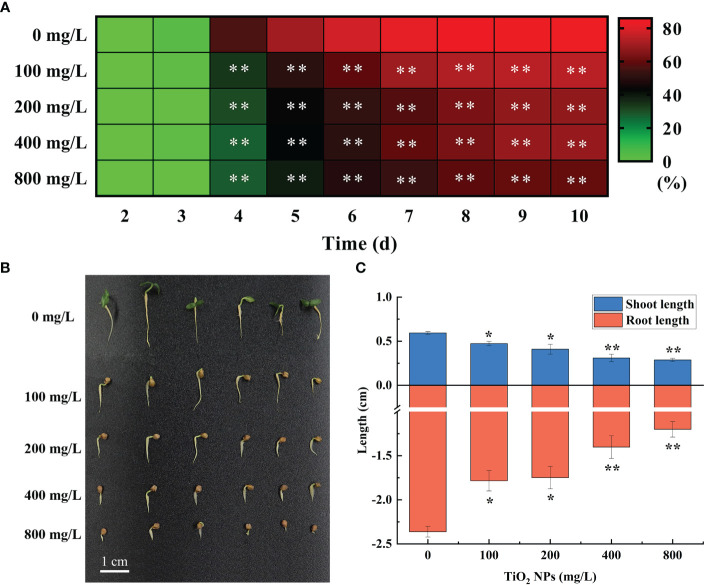
Effects of different concentrations of TiO_2_ NPs on mulberry seed germination. **(A)** Heat map of seed germination rate. **(B)** Seed growth. **(C)** Seed shoot and root length. * indicates that the difference reaches a significant level (P < 0.05), ** indicates that the difference reaches an extremely significant level (P < 0.01). All data are expressed as mean ± standard deviation (n = 100).

In the seed germination experiment, seeds began to germinate on the third day both in the TiO_2_ NPs treatment group and the control group. From the fourth day to the end of germination, the seed germination rate of treatment group was significantly lower than that of the control group (P < 0.01), and showed a concentration-dependent effect. The higher the concentration of TiO_2_ NPs, the lower the seed germination rate. However, there was no significant difference in seed germination rate between the 200 mg/L and 400 mg/L TiO_2_ NPs treatment groups (P > 0.05) (**Figure 2A**). The germination potential, fresh weight, germination index and vigor index of seeds in the treatment group were significantly (P < 0.05) or extreme significantly (P < 0.01) lower than those in the control group; these findings also exhibited a concentration-dependent effect. The higher the concentration, the more significant the inhibitory effect was (**Table S2**). Further study on the growth characteristics of mulberry seedlings revealed that the TiO_2_ NPs in all treatment groups had inhibitory effects on the growth of the shoot and root of mulberry seedlings, and the higher the concentration, the more obvious the inhibitory effect was. As such, the shoot lengths of seedlings in the 100 mg/L and 200 mg/L treatment groups were significantly lower than those in the control group, by 20.33% and 31.08% (P < 0.05), respectively. Furthermore, the root length was significantly lower in the 100 mg/L and 200 mg/L treatment groups compared to in the control group, by 24.44% and 25.94% (P < 0.05), respectively. The shoot lengths identified in the 400 mg/L and 800 mg/L treatment groups were also significantly lower than in the control group, by 47.90% and 51.66% (P < 0.01), respectively, and the root length was significantly lower when comparing the same groups, by 40.61% and 49.18% (P < 0.01), respectively (**Figures 2B, C**). In conclusion, treatment with TiO_2_ NPs can inhibit mulberry seed germination and seedling growth. Broadly, the higher the concentration of TiO_2_ NPs, the more significant the inhibition.

### 3.3 Effects of TiO_2_ NPs on root and leaf tissues of mulberry seedlings

**Figure 3:**
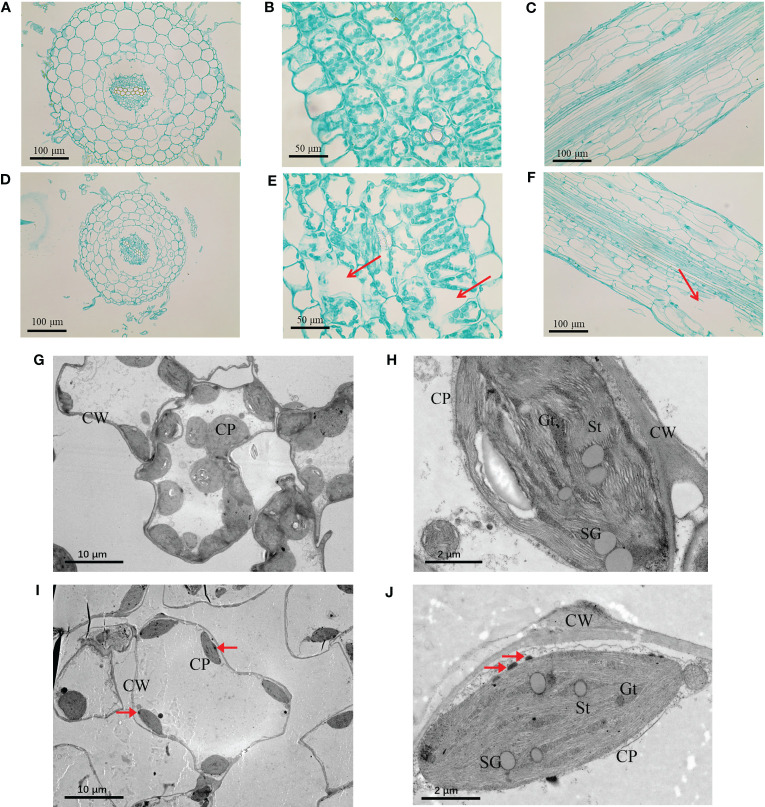
Tissue structure and chloroplast ultrastructure of mulberry seedlings in 400 mg/L TiO_2_ NPs group and control group. **(A)** Root tip cross section of control group. **(B)** Root tip cross section of control group. **(C)** Leaf cross section of control group. **(D)** Root tip cross section of treatment group. **(E)** Root tip cross section of treatment group. **(F)** Leaf cross section of treatment group. **(G, H)** Chloroplast ultrastructure of control group. **(I, J)** Chloroplast ultrastructure of treatment group. CP represents chloroplast, CW represents cell wall, SG represents starch granule, GT represents grana thylakoid, St represents matrix thylakoid, and red arrow represents TiO_2_ NPs suspected substance.

**Figure 4:**
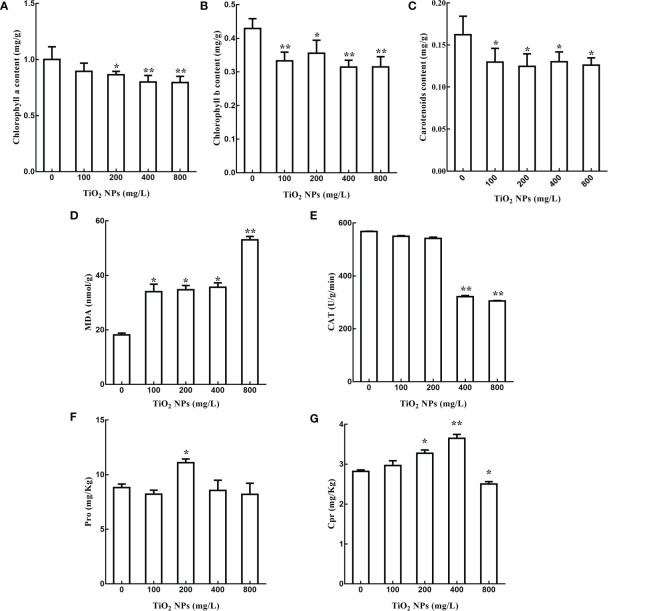
Effects of different concentrations of TiO_2_ NPs on photosynthetic pigments and physiological indexes of stress resistance of mulberry seedlings. **(A)** Change of chlorophyll a content. **(B)** Change of chlorophyll b content. **(C)** Change of carotenoid content. **(D)** Malondialdehyde content. **(E)** Catalase content. **(F)** Proline content. **(G)** Whole protein content. * indicates that the difference reaches a significant level (P < 0.05), ** indicates that the difference reaches an extremely significant level (P < 0.01). All data are expressed as mean ± standard deviation (n = 10).

In order to explore the effects of TiO_2_ NPs on the tissue structure of mulberry seedlings, root and leaf tissue sections were prepared. In analysis of these samples, we revealed that the root tip diameter of mulberry seedlings in the TiO_2_ NPs treatment groups were significantly smaller than in the control group (**Figures 3A, D**), and there were large vacuoles (Red arrow in **Figures 3B, E**). Furthermore, there showed a clear structure damaged in the root of TiO_2_ NPs treatment group (Red arrow in **Figures 3C, F**). Additional TEM observation of the leaves revealed that the number and volume of chloroplasts decreased (**Figures 3G, I**), the cell wall became thinner, starch granules became smaller, and the structures of grana thylakoids and stromal thylakoids were destroyed in the TiO_2_ NPs treatment group (**Figures 3H, J**). Moreover, the content of Chlorophyll a, Chlorophyll b and Carotenoids decreased at 14 days after TiO_2_ NPs treatment in all tested concentrations groups, and all showed significant differences except that the Chlorophyll a in the 100 mg/L treatment group. (**Figures 4A–C**). In conclusion, exposure to TiO_2_ NPs may be not conducive to the growth and photosynthesis of mulberry seedlings resulted in the damage of the root tip and leaf structure.

### 3.4 Effect of TiO_2_ NPs on stress resistance of mulberry seedlings

The growth of mulberry seedlings was inhibited by TiO_2_ NPs, then whether the stress resistance of mulberry seedlings was affected is of interest to us. In plants, malondialdehyde concentration reflects the degree of membrane lipid peroxidation, while catalase is related to drought resistance, salt resistance and oxidation resistance, proline levels are correlated with osmotic stress resistance, and soluble protein concentration is linked to enzymatic activity (Trovato et al., 2008; Gomes et al., 2017; Wu et al., 2017). In order to explore the effects of TiO_2_ NPs on the stress resistance of mulberry seedlings, the levels of malondialdehyde, catalase, proline and soluble protein were measured. The results revealed that the contents of malondialdehyde in 100 mg/L, 200 mg/L and 400 mg/L treatment groups increased by 87.70%, 91.36% and 96.57% respectively compared with the control group (P < 0.05). Moreover, the malondialdehyde concentration in the 800 mg/L treatment group increased by 192.19% compared with the control group, which showed extremely significant level (P < 0.01) (**Figure 4A**). However, there was no significant difference in the levels of catalase both in the 100 mg/L and 200 mg/L treatment groups compared to the control group (P > 0.05), while the content of catalase in the 400 mg/L and 800 mg/L treatment groups decreased by 43.27% and 46.22%, respectively (**Figure 4B**). The proline levels in the 200 mg/L treatment group increased by 25.97% compared to the control group, and the difference reached a significant level (P < 0.05). Conversely, there was no significant difference between other treatment groups and the control group in terms of proline concentration (P > 0.05) (**Figure 4C**). Finally, the soluble protein levels in the 200 mg/L and 400 mg/L treatment groups were increased by 16.11% and 28.68%, respectively, both reached significant level compared with the control group, while the soluble protein concentration in the 800 mg/L treatment group decreased by 11.49% compared with the control group, this difference also reached a significant level (P < 0.05). There was no significant difference between the 100 mg/L treatment group and the control group in terms of soluble protein level (P > 0.05) (**Figure 4D**). In conclusion, different concentrations of TiO_2_ NPs altered the levels of malondialdehyde, catalase, proline and soluble protein in mulberry seedlings and affected the stress resistance of those seedlings.

### 3.5 Absorption and transport of TiO_2_ NPs and metal elements in plants

**Figure 5:**
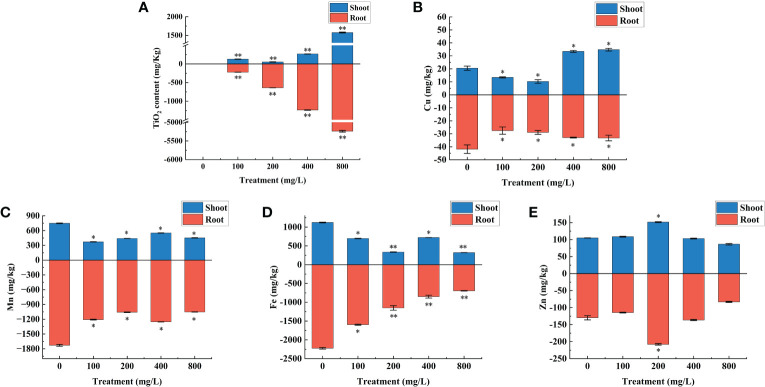
Effects of different concentrations of TiO_2_ NPs on the contents of TiO_2_ and metal elements in mulberry seedlings. **(A)** TiO_2_ content. **(B)** Cu content. **(C)** Mn content. **(D)** Fe content. **(E)** Zn content. * indicates that the difference reaches a significant level (P < 0.05), ** indicates that the difference reaches an extremely significant level (P < 0.01). All data are expressed as mean ± standard deviation (n = 10).

Trace metal elements are closely related to plant growth and development (Dimkpa et al., 2015), so the effect of TiO_2_ NPs on the levels of common essential metal elements in mulberry seedlings were studied. Whether TiO_2_ NPs can be absorbed and transported by mulberry seedlings was determined first. The results revealed that TiO_2_ NPs were not detected in the control group, but were detected in the shoots and roots of the treatment group, and the content of TiO_2_ NPs in the root increased with increasing exposure concentration (**Figure 5A**). The metal elements detection results revealed that the Cu concentration in the root of each treatment group was significantly lower than in the control group (P < 0.05). Furthermore, The Cu content in the shoot varies with the treatment concentration of TiO_2_ NPs. There were significantly lower in both the 100 mg/L and 200 mg/L treatment groups than in the control group (P < 0.05). However, there were significantly higher in the 400 mg/L and 800 mg/L treatment groups than in the control group (P < 0.05) (**Figure 5B**). The concentrations of Mn and Fe in the shoot and root of each treatment group were significantly lower than in the control group (P < 0.05) (**Figures 5C, D**). The Zn levels in the shoot and root of the 200 mg/L treatment group were significantly higher than in the control group (P < 0.05), and there existed no significant difference between the other treatment groups and the control (P > 0.05) (**Figure 5E**). In conclusion, mulberry seedlings can absorb TiO_2_ NPs and transport them from their root to their shoots, subsequently affecting the absorption of metal elements Fe, Mn, Cu and Zn.

### 3.6 Gene ontology and Kyoto encyclopedia of genes and genomes pathway analysis of differentially transcribed genes

To further explore the toxicological mechanism of TiO_2_ NPs in mulberry seedlings, transcriptomic technology was utilized to construct differential transcriptional gene libraries. GO clustering and KEGG pathway analyses were performed.

**Figure 6:**
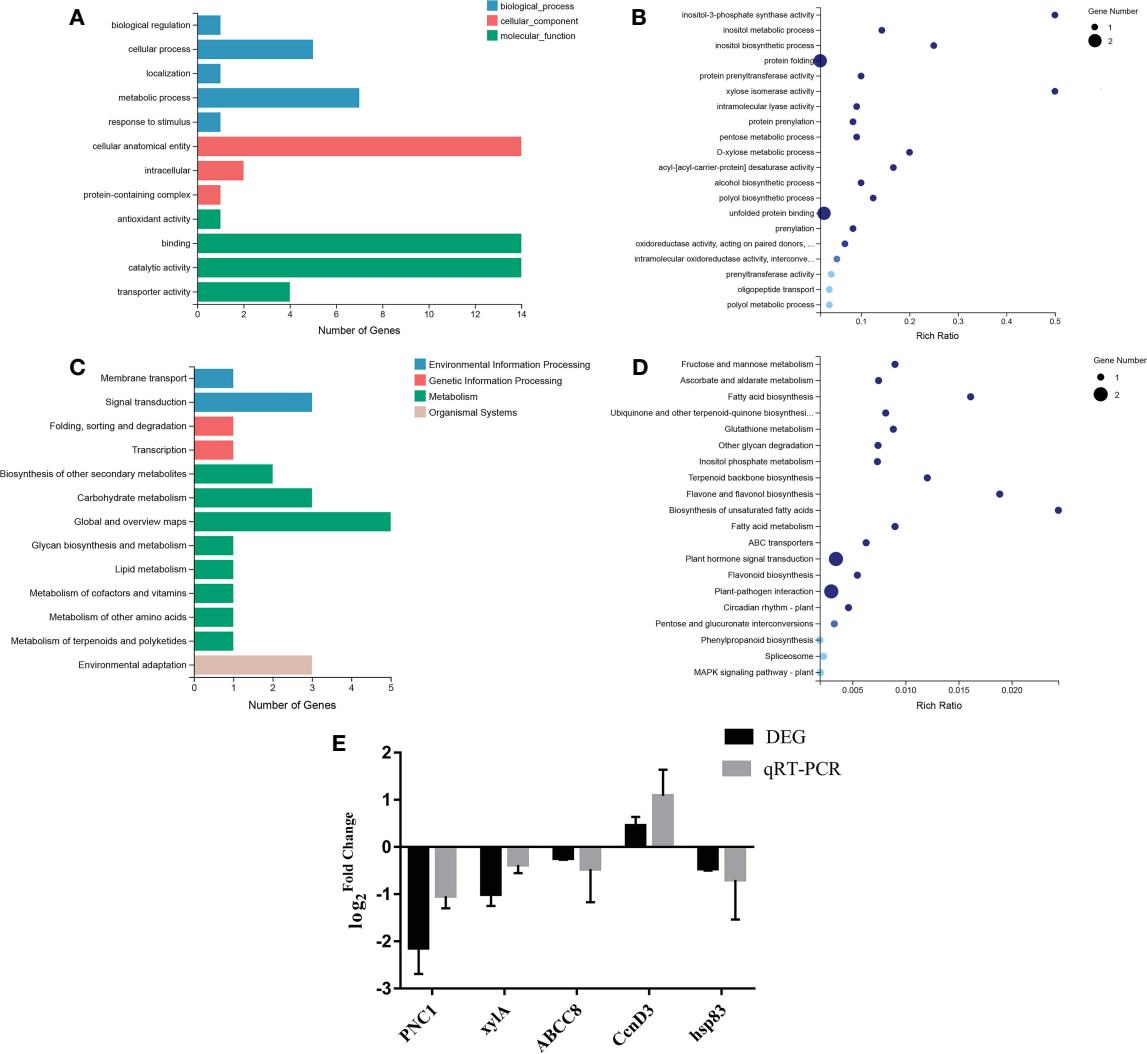
GO analysis, KEGG pathway analysis and qRT-PCR verification. **(A)** GO classification. **(B)** GO enrichment. **(C)** KEGG pathway classification. **(D)** KEGG pathway enrichment. **(E)** qRT-PCR validation of differentially transcribed genes.

GO terms included biological process, cellular component and molecular function. GO classification revealed that biological process genes with differential transcription were mainly enriched within biological regulation, cellular process, localization, metabolic process and stress response, with 1, 5, 1, 7 and 1 differentially expressed genes, respectively. These genes were mainly enriched in the anatomical body of cells, intracellular space or in protein-containing complexes, with 14, 2 and 1 genes, respectively. The molecular function of differentially transcribed genes mainly enriched in antioxidant activity, binding activity, catalytic activity and transport activity, with 14, 14 and 4 genes involved in these functions, respectively (**Figure 6A**). Additional GO enrichment analysis revealed that protein folding and unfolded protein binding were enriched terms for two of the differentially transcribed genes, with a relatively low enrichment rate. The remaining terms were enriched for one differentially transcribed gene, for this gene the enrichment rates for inositol-3-phosphate synthase activity and xylose isomerase activity were relatively high (**Figure 6B**).

The KEGG pathway of differentially transcribed genes mainly involved four branches, including environmental information processing, genetic information processing, metabolism, and organic systems. The environmental information processing genes were mainly enriched in membrane transport (1 gene) and signal transduction (3 genes). In genetic information processing differentially expressed genes were mainly enriched in folding, classification, degradation and transcription, each represented by 1 gene. Meanwhile, differentially transcribed genes were mainly enriched in biosynthesis of secondary metabolites (2 genes), carbohydrate metabolism (3 genes), global and overview maps (5 genes), polysaccharide biosynthesis and metabolism (1 gene), lipid metabolism (1 gene), metabolism of cofactors and vitamins (1 gene), metabolism of other amino acids (1 gene), metabolism of terpenoids and polyketones (1 gene). Finally, differentially expressed genes in organic systems were mainly enriched in environmental adaptation (3 genes) (**Figure 6C**). Further KEGG enrichment analysis of differentially transcribed genes revealed that plant hormone signal transduction and plant-pathogen interaction were both enriched in two differentially transcribed genes with relatively low enrichment rate. Other pathways were enriched in one differential transcription gene, among these the enrichment rate of biosynthesis of unsaturated fatty acids was relatively high (**Figure 6D**).

### 3.7 Differentially transcribed genes screening and verification

In this study, six genes related to energy synthesis and transportation, protein metabolism and response to stress were screened from the differential transcriptional gene library. Furthermore, the transcriptional levels of genes related to energy synthesis and transportation were down-regulated. Among protein metabolism-related genes, *disease resistance protein RPM1* and *RNA-binding protein 24* were up-regulated, while other genes were down-regulated. Among stress-responsive genes, *Serine/threonine-protein kinase HT1* and *Morus Notabilis Cyclin-D3-1* were up-regulated, while other genes were down-regulated (**Table S3**). To further verify the reliability of the transcriptome data, five differentially transcribed genes were randomly selected for qRT-PCR analysis. The variability of the transcriptome level trend was consistent with the transcriptome sequencing results, indicating that the sequencing results were highly reliable (**Figure 6E**). In conclusion, TiO_2_ NPs can attenuate the energy synthesis and transport capacity of mulberry seedlings resulting in disruption to protein metabolism and stress responses.

### 3.8 Metabolomics analysis

**Figure 7:**
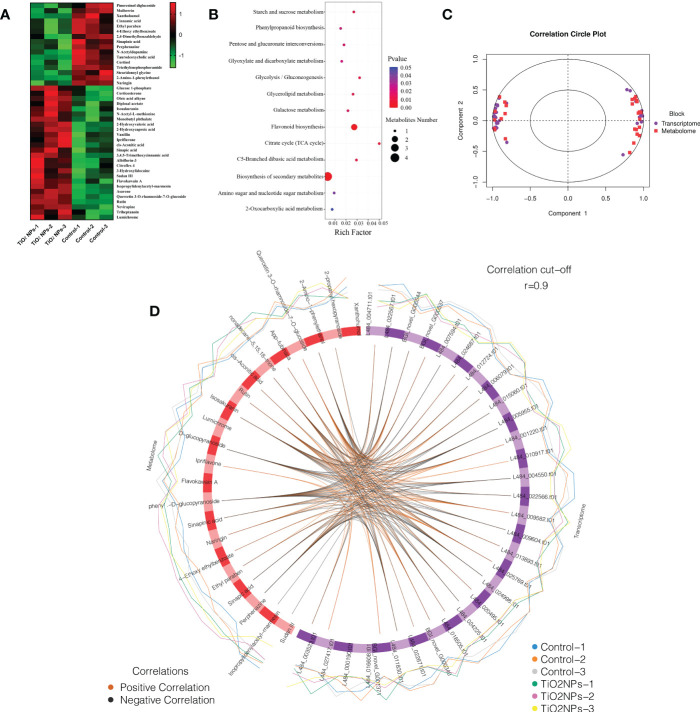
Differential metabolite analysis and correlation analysis of transcriptome and metabolomics. **(A)** Heat map of differential metabolites. **(B)** Bubble map of differential metabolite enrichment analysis. **(C)** Concentric circle diagram of the correlation between differentially transcribed genes and differential metabolites. Each point in the circle represents a gene and each square represents a metabolite, and the relationship between differentially transcribed genes and differential metabolites was determined by projection on the first and second principal components. The length of the line to the differential metabolites and differentially transcribed genes, starting from the center of the circle, is positively correlated with the correlation. Positive values represent positive correlations and negative values represent negative correlations. **(D)** Circos map of the correlation between differential genes and differential metabolites. The lines in the circle represent the correlation coefficient between differential genes and differential metabolites greater than or equal to 0.9. The blue and orange peripheral curves represent the expression of differential metabolites and differential genes in the two groups of samples.

Metabolomics technology enables the qualitative and quantitative analysis of all low-molecular-weight metabolites in organisms and the screening of key metabolic pathways (Goodacre, 2004; Dehaven et al., 2010). To the effects that exposure to TiO_2_ NPs had on mulberry seedling metabolism, metabolomics technology was used to measure the expression characteristics of a multitude of differential metabolites. A total of 42 differential metabolites were annotated, and there were more up-regulated differential metabolites than down-regulated differential metabolites. The expression levels of 16 differential metabolites, including Pinoresinol diglucoside, Mulberrin and Xanthohumol were down-regulated. Conversely, 26 differential metabolites, including Glucose 1-phosphate, Corticosterone and Oleic acid alkyne, were up-regulated (**Figure 7A**). Further enrichment analysis of differential metabolites revealed that Glucose 1-phosphate, cis-Aconitic acid, Sinapic and Isosakuranin were annotated to the biosynthesis of secondary metabolites pathway. Xanthohumol and Isosakuranin were annotated to the flavonoid biosynthesis pathway. Interestingly, the compound of Isosakuranin was identified for the first time in *Morus alba*. There were eleven differential metabolites annotated to eleven different metabolism pathways, such as starch and sucrose metabolism pathway, phenylpropanoid biosynthesis, pentose and glucuronate interconversions et al. In conclusion, the enrichment rate of differential metabolites in terms of biosynthesis of secondary metabolites was relatively low. Conversely, the enrichment rate of differential metabolites in the citrate cycle (TCA cycle) pathway was relatively high (**Figure 7B**).

### 3.9 Correlation analysis of transcriptome and metabonome

In this study, correlation analysis of transcriptome and metabonome revealed that most differentially transcribed genes were strongly correlated with differential metabolites (**Figure 7C**). The same differential metabolite could be correlated with multiple differentially transcribed genes. Interestingly, 2-propanyl hexopyranoside was correlated with eleven differentially transcribed genes, 2-Amino-1-phenylethanol correlated with nine differentially transcribed genes, Xanthohumol was correlated with eight differentially transcribed genes, Flavokawain A was correlated with seven differentially transcribed genes, Sinapic acid was correlated with six differentially transcribed genes, and (6,6-dimethylbicyclo[3.1.1]hept-2-yl) methyl 6-O-[(2R,3R,4R)-3,4-dihydroxy-4-(hydroxymethyl) tetrahydro-2-furanyl]-β-D- glucopyranoside was correlated with six differentially transcribed genes (**Figure 7D**). Meanwhile, each differentially transcribed gene could be correlated with multiple differential metabolites, for example L484_024998.t01, L484_004711.t01, L484_011830.t01, L484_005955.t01, L484_009604.t01, BGI_novel_G001371, and L484_000190.t01 was correlated with night, eight, seven, six, six, six, and five differential metabolites, respectively (**Figure 7D**; **Table S4**).

## 4 Discussion

The biological and environmental safety of nanomaterials is of increasing concern. The mulberry is used as feed for silkworms. In addition, it also is widely used in food, healthcare, and ecological management. However, there are no studies about the effects of nanomaterials on mulberry plants have been reported to date. In the present study, we identified that TiO_2_ NPs reduce the germination rate of mulberry seeds, inhibit their shoot and root growth (**Figure 2**), and exhibit toxic effects that attenuate the growth of mulberry seedlings similar to those reported in graminaceous maize and the solanaceous tobacco crops (Castiglione et al., 2011; Frazier et al., 2014). We speculate that the toxic effects of TiO_2_ NPs on mulberry seedlings may be caused by three aspects.

Firstly, the toxicity of TiO_2_ NPs may be due to disruption of the root tissue structure of mulberry seedling, whereby the NPs appear to inhibit the formation of iron films on the root surface. NPs of small particle size and high surface activity can be adsorbed through the plant root surface (Wang et al., 2012b; Tripathi et al., 2017), and the Ti was detected in both roots and shoots of mulberry seedlings (**Figure 5A**) indicating that TiO_2_ NPs could be absorbed and transported by the seedling. Analysis of mulberry root tissue sections revealed that TiO_2_ NPs had a destructive effect on the tissue structure of mulberry seedling roots (**Figures 3A, B, D, E**). Some studies have reported that the destruction of plant root structure by nanoparticles may be the direct cause of toxicity (Hu et al., 2022). Furthermore, we observed that TiO_2_ NPs altered the Cu, Mn, Fe, and Zn contents in shoots and roots of mulberry seedlings, resulting in a decrease of both Mn and Fe in the roots (**Figures 5B–E**). Mn and Fe are the primary components of the iron film of the plant root surface (Tang et al., 2021), and the decrease in concentration of these ions is detrimental to the formation of the iron film, which in turn affects the uptake of Cu and Zn by mulberry seedlings resulting in the disruption of the homeostasis of metal ions and subsequent toxicity (Rico et al., 2014; Dimkpa et al., 2015; Stewart et al., 2015).

Secondly, the effect of TiO_2_ NPs on the photosynthesis of mulberry seedlings may contribute to their toxicity. TiO_2_ NPs translocated to the shoots of mulberry seedlings and lead to damage to the leaf structure. Exposure to TiO_2_ NPs caused a reduction of chloroplasts in leaves as well as structural damage (**Figure 3**) and a decrease in chlorophyll a, chlorophyll b and carotenoid contents (**Figures 4E, F)**, resulting in abnormal photosynthesis. Since TiO_2_ NPs affect the uptake of metal ions by mulberry seedlings (**Figures 5B–E**), the combined effects would compound the attenuation of photosynthesis (Hansch and Mendel, 2009). Furthermore, in the transcriptome analysis of mulberry seedlings, a total of six differentially transcribed genes related to energy synthesis and transport were associated with decreased expression (**Table S3**), indicating that TiO_2_ NPs hinder energy synthesis and metabolism in mulberry seedlings, further demonstrating that TiO_2_ NPs are detrimental to photosynthesis, which is similar to the results of toxicity studies in the crucifer *Arabidopsis thaliana* (Szymańska et al., 2016).

Ultimately, TiO_2_ NPs affected the stress resistance of mulberry seedlings. It was identified that different concentrations of TiO_2_ NPs increased malondialdehyde content, and that low concentrations of TiO_2_ NPs increased proline and soluble protein content, while high concentrations of TiO_2_ NPs decreased catalase and soluble protein content (**Figure 4**). Overall, this indicated that TiO_2_ NPs affected the stress resistance of mulberry seedlings. In addition, six differentially transcribed genes correlated with stress responses were screened through transcriptome analyses. Of these, the expression of genes related to stress resistance, metal metabolism, and abscisic acid signaling were down-regulated, the expression of genes related to cell volume regulation and cellular autophagy was up-regulated (**Table S3**), further demonstrating that TiO_2_ NPs affect the stress resistance of mulberry seedlings, similar to the results of a related study in rice (Wu et al., 2017).

Currently, there are few studies reporting the phytotoxicity of TiO_2_ NPs to plants at both transcriptome and metabonome levels (Wu et al., 2017). Due to the mulberry genome database is not perfect, many genes have not been identified. In the present study, only six differentially transcribed genes related to protein metabolism were screened, of which, four differential genes were down-regulated and two were up-regulated (**Table S3**). In addition, 42 differential metabolites were annotated in the results of the mulberry seedling metabolome, of which, 16 differential metabolites that were down-regulated and 26 that were up-regulated (**Figure 7A**). These results indicated that TiO_2_ NPs affected the expression of metabolism-related genes, resulting in disordered metabolite expression. To further investigate the regulatory relationship between differentially transcribed genes and differential metabolites, correlation analysis was performed and a strong correlation was identified between specific differentially transcribed genes and differential metabolites (**Figure 7C**). For example, Sinapic acid, which is a natural herbal compound widely found in the plant kingdom with antioxidant, anti-inflammatory and antibacterial effects, has been shown to attenuate various chemically induced toxicities (Chen, 2016; Pandi and Kalappan, 2021), while high concentrations of Sinapic acid are cytotoxic (Han et al., 2022). The metabolic pathways phenylpropanoid biosynthesis and biosynthesis of secondary metabolites were both annotated to Sinapic acid (**Figure 7B**), and its expression was up-regulated (**Figure 7A**). Genes L484_004550.t01, L484_ 005955.t01, L484_015060.t01, L484_024225.t01 and L484_024998.t01 were negatively correlated or positively correlated with L484_006079.t01 (**Figure 7D**), indicating that L484_004550.t01, L484_005955.t01, L484_ t01, L484_024225.t01, L484_024998.t01, and L484_006079.t01 were correlated (**Figure 7D**). Cis-Aconitic acid is an intermediate product of the tricarboxylic acid cycle, it possesses anti-inflammatory effects (Oliveira et al., 2021). Many metabolic pathways were identified as being up-regulated through correlation with increased expression of cis-Aconitic acid (**Figure 7A**), including glyoxylate and dicarboxylate metabolism, the citrate cycle (TCA cycle), C5-branched dibasic acid metabolism, biosynthesis of secondary metabolites and 2-Oxocarboxylic acid (**Figure 7B**), with the genes BGI_novel_G000340, BGI_novel_G001371 and L484_ 027417.t01 (**Figure 7D**), being positively correlated with each. These systems together regulate the expression of cis-Aconitic acid expression.

Xanthohumol is an important isopentenyl flavonoid with antioxidant, antibacterial and anti-inflammatory effects (Lv et al., 2017; Harish et al., 2021). Flavonoid biosynthesis was annotated to Xanthohumol (**Figure 7B**), and its expression was downregulated (**Figure 7A**), which negatively correlated with genes BGI_novel_G000544, L484_001220.t01, L484_009582.t01, L484_010917.t01, and L484_ 012724.t01, but positively correlated with L484_011830.t01, L484_016608.t01, and L484_018505.t01 (**Figure 7D**), indicating that a total of eight genes regulate Xanthohumol’s expression through flavonoid biosynthesis. Furthermore, Isosakuranin was first isolated from the fruit of *Paliurus ramosissimus* and later from the bark of *Populus tomentosa* (Wu et al., 2021). In this study the compound was identified for the first time in *Morus alba*. The results of the transcriptome and metabonome correlation analysis showed that both flavonoid biosynthesis and biosynthesis of secondary metabolites were annotated to Isosakuranin (**Figure 7B**), and its expression was up-regulated after TiO_2_ NP exposure (**Figure 7A**). As such, the genes L484_004711.t01, L484_ 011830.t01 and L484_024998.t01 were negatively correlated with Isosakuranin but BGI_novel_G001371 (**Figure 7D**) were positively correlated, indicating that a total of three genes, L484_004711.t01, L484_011830.t01 and L484_024998.t01, were expressed differentially to regulate the expression of Isosakuranin. In summary, differentially transcribed genes were observed to regulate the expression of metabolites including Sinapic acid, cis-Aconitic acid, Xanthohumol and Isosakuranin through metabolic pathways and affect the resistance of mulberry seedlings to TiO_2_ NPs.

**Figure 8:**
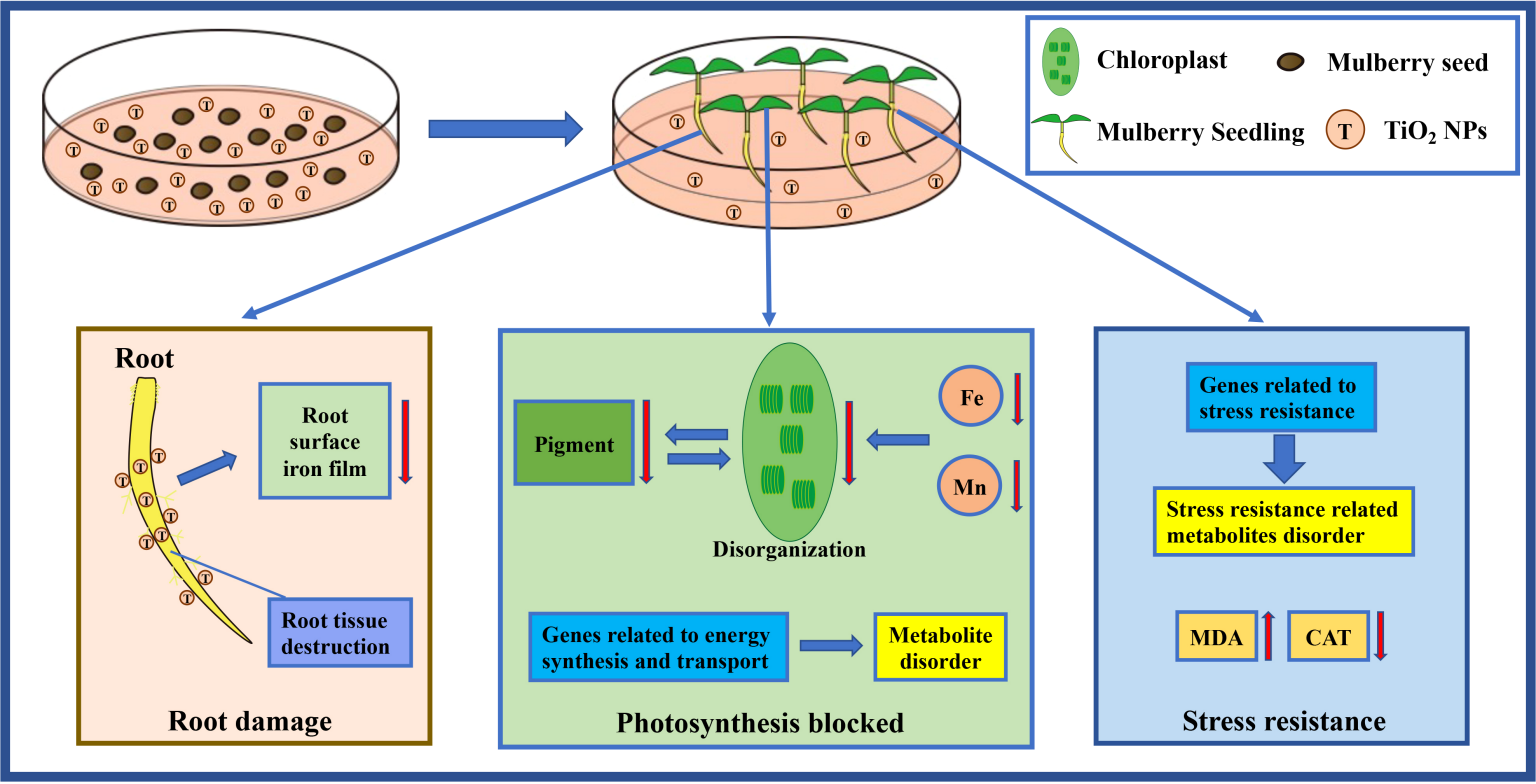
The pattern diagram of toxicity mechanism of TiO_2_ NPs to mulberry seedlings.

In summary, the TiO_2_ NPs caused damage to the root tissue structure of mulberry seedlings, which was detrimental to the formation of iron film on the root surface. Consequently, TiO_2_ NPs hinder photosynthesis and affect the resistance of mulberry seedlings. Thus, we propose a pattern herein that TiO_2_ NPs exhibit toxicity in mulberry seedlings (**Figure 8**).

Furthermore, the suspected TiO_2_ NPs were observed on the chloroplast surface of mulberry seedlings (**Figures 3G–J**). The puzzle is how does TiO_2_ NPs enter cells of the mulberry seedling. The TiO_2_ NPs used in this study had a particle size concentrated within the range of 12-18 nm. It had been reported that only molecules smaller than 10 nm can freely pass through the plant cell wall (Carpita et al., 1979), so we speculated that it is unlikely that TiO_2_ NPs directly penetrate the cell wall of mulberry seedlings. Moreover, it was identified that the cytoderm of mulberry seedlings became thinner after treatment with TiO_2_ NPs (**Figures 3I, J**). There had similar report that silica particles in the size range of 50 nm to 1 µm can all taken up by cell, although the endocytosis pathway was dependent on the particle geometry (Kersting et al., 2020). Thus, we hypothesize that TiO_2_ NPs disrupt the cell wall of mulberry seedlings resulting in increased permeability, and this enables the TiO_2_ NPs to more easily penetrate the cells. However, the precise mechanism needs to be further investigated.

## 5 Conclusions

In summary, we revealed that TiO_2_ NPs could be absorbed by the root system and be transported to the shoot of mulberry seedlings, resulting in damage to the root tip and leaf tissue. Further, the metal ion homeostasis was destroyed, photosynthesis was weakened, and the resistance of mulberry seedlings was destroyed. Overall, TiO_2_ NPs were detrimental to seed germination and seedling growth with significant toxic effects exhibited by mulberry seedlings in a dose-dependent manner. In addition, treatment with TiO_2_ NPs altered the transcript levels of a multitude of genes related to energy synthesis and transport, protein metabolism, and response to stress, and thus revealing significant alterations to the metabolome profile of mulberry seedlings in the short term.

## Data availability statement

The datasets presented in this study can be found in online repositories. The names of the repository/repositories and accession number(s) can be found below: NCBI BioProject accession number: PRJNA947319.

## Author contributions

KX, DY and CQ conceived and designed the experiments. QL, YW, and DH performed the experiments. DY, XY, and KC analyzed the data. KX, DY, and QL wrote the manuscript. All authors contributed to the article and approved the submitted version.
