# Supplementary material for: Combined analysis of transcriptomics and metabolomics on the cumulative effect of nano-titanium dioxide on mulberry seedlings
Source: Front Plant Sci. 2023 Jun 14;14:1175012. doi: 10.3389/fpls.2023.1175012 (PMC10301732; doi:10.3389/fpls.2023.1175012)
Supplement: Supplementary file 1 [file DataSheet_1.docx]

Supplementary Material

Combined analysis of transcriptomics and metabonomics on the cumulative effect of nano-titanium dioxide on mulberry seedlings

Dongliang Yu^1^, Qingyu Lu^1^, Yuting Wei^1^, Di Hou^1^, Xingcan Yin ^1^, Kunpei Cai^1^, Changyu Qiu^3^, Kaizun Xu^1,2*^

^1^College of Agriculture, Guangxi University, Nanning, Guangxi 530004, PR China

^2^Guangxi Key Laboratory of Agro-Environment and Agric-Products Safety, National Demonstration Center for Experimental Plant Science Education, College of Agriculture, Guangxi University, Nanning, Guangxi 530004, PR China

^3^Sericulture Technology Promotion Station of Guangxi Zhuang Autonomous Region, Nanning, Guangxi 530007, PR China

*** Correspondence:**Corresponding Author: Kaizun Xu, Department of Plant Protection, College of Agriculture, Guangxi University, Nanning 530004, PR China
[kzxu@gxu.edu.cn](mailto:kzxu@gxu.edu.cn)

# Supplementary Data

**
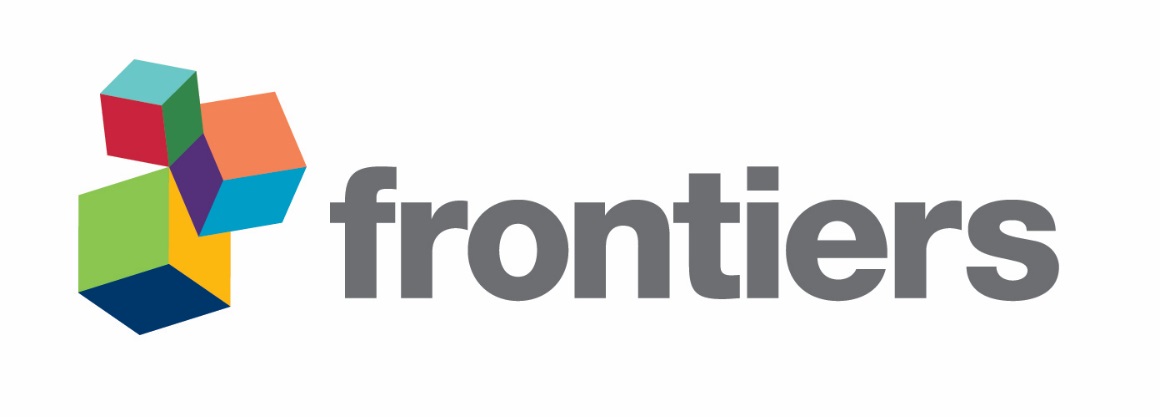
**

**Text S1.** The calculation formula of germination index.

**Text S2.** The calculation formula of photosynthetic pigment content calculation.

**Fig S1.** The pattern diagram of toxicity mechanism of TiO_2_ NPs to mulberry seedlings.

**Table S1.** Primers used in this study.

**Table S2.** Germination indexes of different concentrations of TiO_2_ NPs.

**Table S3.** Screening of differentially transcribed genes

**Table S4.** Correlation analysis of transcriptomic and metabolomic data.

**Text S1. The calculation formula of germination index.**

Germination rate (%) = germination number of seeds/total number of seeds ×100% (1)

Germination potential (%) = number of germinated seeds at germination peak/total number of tested seeds ×100% (2)

Germinating index = ∑ (germinating number at time t/t) (3)

Vigor index = fresh weight of seedlings ×∑ (germinated number at time t/t) (4)

**Text S2. The calculation formula of photosynthetic pigment content calculation.**

[Chlorophyll a] = 13.95A665 – 6.88A649 (5)

[Chlorophyll b] = 24.96A649 – 7.32A665 (6)

[Carotenoids] = [1000A470 – 2.05(Chlorophyll a) – 114.8(Chlorophyll b)]/245 (7)

**Table S1. Primers used in this study.**

| Gene name | Primer sequences (5’ - 3’) | Length of product (bp) |
| --- | --- | --- |
| *β-actin* | F：TCCGATGCCCAGAAGTCCTC | 189 |
|  | R：TGATCTCCTTGCTCATCCTGTCT |  |
| *PNC1* | F：AGTCGAATTTGGTTCCCGCT | 136 |
|  | R：TGCTTCACCCAGTCCACAAA |  |
| *XylA* | F：TGGACACCGACGCATCATTA | 151 |
|  | R：GTAACTGTCCCTGGAGCCAC |  |
| *ABCC8* | F：TGGACACCGACGCATCATTA | 151 |
|  | R：GTAACTGTCCCTGGAGCCAC |  |
| *CcnD3* | F：TCCCAAGAGAAAGAAGCACGA | 175 |
|  | R：GCCTTCCTCAATCAAGCCAC |  |
| *Hsp83* | F：TCGGTGTCGGGTTTTACTCG | 159 |
|  | R：TCTTGGTTCCTCTGCCCAAC |  |

**Table S2. Germination indexes of different concentrations of TiO_2_ NPs. *Indicates that the difference reaches a significant level (P < 0.05), ** indicates that the difference reaches a very significant level (P < 0.01).**

| Germination index | 0 mg/L | 100 mg/L | 200 mg/L | 400 mg/L | 800 mg/L |
| --- | --- | --- | --- | --- | --- |
| Germination potential (%) | 56.3±1.76 | 33.7±1.86** | 30.3±0.88** | 27.0±1.73** | 27.7±0.88** |
| Fresh weight (g) | 0.017±0.0002 | 0.014±0.0007* | 0.013±0.0008** | 0.014±0.0008* | 0.012±0.0005** |
| Germination index | 82.4±2.22 | 63.7± 1.55** | 55.7± 2.17** | 55.4±2.28** | 50.7±1.16** |
| Vitality index | 1.4±.055 | 0.9±.030** | 0.7± .048** | 0.8±0.074** | 0.6±0.024** |

**Table S3. Screening of differentially transcribed genes**

| Name | Sequence ID | Q value | log_2_(TiO_2_ NPs/Control) |
| --- | --- | --- | --- |
| Energy synthesis and transportation |  |  |  |
| *nicotinamidase 1* | L484_000190.t01 | 3.67E-05 | -1.731976527 |
| *ABC transporter C family member 8* | L484_004550.t01 | 2.18E-06 | -0.80898406 |
| *NADP-dependent alkenal double bond reductase P2* | L484_018505.t01 | 1.18E-05 | -0.805298024 |
| *putative transporter MCH1* | *L484_022567.t01* | *4.33E-05* | *-1.185756357* |
| *Xylose isomerase* | *L484_022871.t01* | *1.04E-22* | *-2.897943211* |
| *L-ascorbate peroxidase 3* | L484_024998.t01 | 1.20E-05 | -0.518696928 |
| Protein metabolism |  |  |  |
| *desiccation-related protein* | L484_005955.t01 | 3.49E-05 | -1.155742517 |
| *Protein kinase byr2* | [*L484_007594.t01*](https://report.bgi.com/ps/tools/index.html#/home/geneDetail/L484_007594.t01) | *9.94E-06* | *-1.217042195* |
| *Disease resistance protein RPM1* | L484_009582.t01 | 3.84E-05 | 0.753717898 |
| *protein DETOXIFICATION 19* | L484_009604.t01 | 3.01E-06 | -0.654981094 |
| *RNA-binding protein 24* | [L484_012724.t01](https://report.bgi.com/ps/tools/index.html#/home/geneDetail/L484_012724.t01) | 2.03E-05 | 0.829112256 |
| *Heat shock protein 83* | L484_024225.t01 | 2.89E-08 | -1.411219149 |
| Response to stress |  |  |  |
| *Serine/threonine-protein kinase HT1* | L484_006079.t01 | 2.31E-05 | 0.721787463 |
| *DnaJ homolog subfamily B member 13* | L484_015060.t01 | 2.28E-05 | -0.968603771 |
| *Inositol-3-phosphate synthase* | [L484_016608.t01](https://report.bgi.com/ps/tools/index.html#/home/geneDetail/L484_016608.t01) | 1.23E-05 | -1.374791371 |
| *Protein farnesyl transferase subunit beta* | L484_020495.t01 | 5.30E-07 | -0.539253083 |
| *Salicylate O-methyltransferase* | L484_026743.t01 | 3.51E-08 | -1.787969408 |
| *Morus notabilis cyclin-D3-1* | L484_027417.t01 | 4.19E-05 | 1.291867097 |

**Table S4. Correlation analysis of transcriptomic and metabolomic data**

| **No.** | **Gene name** | **GeneID** | **R** | **pvalue** |
| --- | --- | --- | --- | --- |
| **6,12,17-Trihydroxy-16-methyl-8-2,4,14,19-tetraoxahexacyclo-nonadecane-5,15,18-trione related genes** | | | | |
| 1 | Flavonoid 3'-monooxygenase | L484_004711.t01 | -0.94286 | 0.016667* |
| **methyl 6-O-β-D-glucopyranoside related genes** | | | | |
| 1 | ABC transporter C family member 8 | L484_004550.t01 | -0.94286 | 0.016667* |
| 2 | Desiccation-related protein | L484_005955.t01 | -0.94286 | 0.016667* |
| 3 | Serine/threonine-protein kinase HT1 | L484_006079.t01 | 0.942857 | 0.016667* |
| 4 | DnaJ homolog subfamily B member 13 | L484_015060.t01 | -0.94286 | 0.016667* |
| 5 | Heat shock protein 83 | L484_024225.t01 | -0.94286 | 0.016667* |
| 6 | L-ascorbate peroxidase 3 | L484_024998.t01 | -0.94286 | 0.016667* |
| **propanyl hexopyranoside related genes** | | | | |
| 1 | Acyl-[acyl-carrier-protein] desaturase 6 | L484_003523.t01 | 1 | 0.002778** |
| 2 | ABC transporter C family member 8 | L484_004550.t01 | -0.94286 | 0.016667* |
| 3 | Desiccation-related protein PCC13-62 | L484_005955.t01 | -0.94286 | 0.016667* |
| 4 | Serine/threonine-protein kinase HT1 | L484_006079.t01 | 0.942857 | 0.016667* |
| 5 | Protein kinase byr2 | L484_007594.t01 | -0.94286 | 0.016667* |
| 6 | Detoxfication protein 19 | L484_009604.t01 | -0.92763 | 0.007666** |
| 7 | DnaJ homolog subfamily B member 13 | L484_015060.t01 | -0.94286 | 0.016667* |
| 8 | Protein farnesyltransferase subunit beta | L484_020495.t01 | -0.94286 | 0.016667* |
| 9 | Hypothetical protein | L484_022566.t01 | -1 | 0.002778** |
| 10 | Putative transporter MCH1 | L484_022567.t01 | -1 | 0.002778** |
| 11 | Heat shock protein 83 | L484_024225.t01 | -0.94286 | 0.016667* |
| **2-Amino-1-phenylethanol related genes** | | | | |
| 1 | ABC transporter C family member 8 | L484_004550.t01 | -0.94286 | 0.016667* |
| 2 | Serine/threonine-protein kinase HT1 | L484_006079.t01 | 0.942857 | 0.016667* |
| 3 | Disease resistance protein RPM1 | L484_009582.t01 | 1 | 0.002778** |
| 4 | Transcription factor bHLH130 | L484_010917.t01 | 0.942857 | 0.016667* |
| 5 | RNA-binding protein 24 | L484_012724.t01 | 1 | 0.002778** |
| 6 | DnaJ homolog subfamily B member 13 | L484_015060.t01 | -0.94286 | 0.016667* |
| 7 | Inositol-3-phosphate synthase | L484_016608.t01 | -0.94286 | 0.016667* |
| 8 | NADP-dependent alkenal double bond reductase P2 | L484_018505.t01 | -0.94286 | 0.01666*7 |
| 9 | Heat shock protein 83 | L484_024225.t01 | -0.94286 | 0.01666*7 |
| **2-Hydroxy-3-phenyl β-D-glucopyranoside related genes** | | | | |
| 1 | Putative receptor protein kinase TMK1 | L484_001220.t01 | 0.942857 | 0.016667* |
| 2 | Flavonoid 3'-monooxygenase | L484_004711.t01 | -0.94286 | 0.016667* |
| 3 | Putative methyltransferase PMT18 | L484_011830.t01 | -0.94112 | 0.005098** |
| 4 | L-ascorbate peroxidase 3 | L484_024998.t01 | -1 | 0.002778** |
| 5 | Topless-related protein 4 | L484_025769.t01 | 0.942857 | 0.016667* |
| **4-Ethoxy ethylbenzoate related genes** | | | | |
| 1 | Homogentisate solanesyltransferase | BGI_novel_G001371 | 0.942857 | 0.016667* |
| 2 | Flavonoid 3'-monooxygenase | L484_004711.t01 | -1 | 0.002778** |
| 3 | Putative methyltransferase PMT18 | L484_011830.t01 | -0.94112 | 0.005098** |
| 4 | L-ascorbate peroxidase 3 | L484_024998.t01 | -0.94286 | 0.016667* |
| **App-fubinaca related genes** | | | | |
| 1 | Morus notabilis protein NRT1 | BGI_novel_G000340 | 0.942857 | 0.016667* |
| 2 | Disease resistance-like protein | BGI_novel_G000537 | -0.94286 | 0.016667* |
| 3 | Nicotinamidase 1 | L484_000190.t01 | -0.98561 | 0.000309** |
| 4 | Uncharacterized | L484_013893.t01 | -0.94286 | 0.016667* |
| 5 | Cyclin-D3-1 | L484_027417.t01 | 0.942857 | 0.016667* |
| **Cinnamic acid related genes** | | | | |
| 1 | Homogentisate solanesyltransferase | BGI_novel_G001371 | 0.942857 | 0.016667* |
| 2 | Flavonoid 3'-monooxygenase | L484_004711.t01 | -1 | 0.002778** |
| 3 | Putative methyltransferase PMT18 | L484_011830.t01 | -0.94112 | 0.005098** |
| 4 | L-ascorbate peroxidase 3 | L484_024998.t01 | -0.94286 | 0.016667* |
| **cis-Aconitic acid related genes** | | | | |
| 1 | Morus notabilis protein NRT1 | BGI_novel_G000340 | 0.942857 | 0.016667* |
| 2 | Homogentisate solanesyltransferase | BGI_novel_G001371 | 0.942857 | 0.016667* |
| 3 | Cyclin-D3-1 | L484_027417.t01 | 0.942857 | 0.016667* |
| **Ethyl paraben related genes** | | | | |
| 1 | Homogentisate solanesyltransferase | BGI_novel_G001371 | 0.942857 | 0.016667* |
| 2 | Flavonoid 3'-monooxygenase | L484_004711.t01 | -1 | 0.002778** |
| 3 | Putative methyltransferase PMT18 | L484_011830.t01 | -0.94112 | 0.005098** |
| 4 | L-ascorbate peroxidase 3 | L484_024998.t01 | -0.94286 | 0.016667* |
| **Flavokawain A related genes** | | | | |
| 1 | Acyl-[acyl-carrier-protein] desaturase 6 | L484_003523.t01 | -0.94286 | 0.016667* |
| 2 | Protein kinase byr2 | L484_007594.t01 | 1 | 0.002778** |
| 3 | Detoxfication protein 19 | L484_009604.t01 | 0.985611 | 0.000309** |
| 4 | Protein farnesyltransferase subunit beta | L484_020495.t01 | 1 | 0.002778** |
| 5 | Hypothetical protein | L484_022566.t01 | 0.942857 | 0.016667* |
| 6 | Putative transporter MCH1 | L484_022567.t01 | 0.942857 | 0.016667* |
| 7 | Xylose isomerase | L484_022871.t01 | 0.942857 | 0.016667* |
| **Ipriflavone related genes** | | | | |
| 1 | Morus notabilis protein NRT1 | BGI_novel_G000340 | 1 | 0.002778** |
| 2 | Nicotinamidase 1 | L484_000190.t01 | -0.92763 | 0.007666** |
| 3 | Cyclin-D3-1 | L484_027417.t01 | 1 | 0.002778** |
| **Isopropylidenylacetyl-marmesin related genes** | | | | |
| 1 | Disease resistance-like protein | BGI_novel_G000537 | 1 | 0.002778** |
| 2 | Nicotinamidase 1 | L484_000190.t01 | 0.985611 | 0.000309** |
| 3 | Homogentisate solanesyltransferase | BGI_novel_G001371 | 0.942857 | 0.016667* |
| 4 | Flavonoid 3'-monooxygenase | L484_004711.t01 | -1 | 0.002778** |
| 5 | putative methyltransferase PMT18 | L484_011830.t01 | -0.94112 | 0.005098** |
| 6 | L-ascorbate peroxidase 3 | L484_024998.t01 | -0.94286 | 0.016667* |
| **Lumichrome related genes** | | | | |
| 1 | Uncharacterized | L484_024687.t01 | 0.942857 | 0.016667* |
| **Mulberrin related genes** | | | | |
| 1 | Uncharacterized | L484_013893.t01 | 0.942857 | 0.016667* |
| 2 | Uncharacterized | L484_024687.t01 | 1 | 0.002778** |
| 3 | Salicylate O-methyltransferase | L484_026743.t01 | 0.942857 | 0.016667* |
| **Naringin related genes** | | | | |
| 1 | Disease resistance-like protein | BGI_novel_G000537 | -0.94286 | 0.016667* |
| 2 | Desiccation-related protein PCC13-62 | L484_005955.t01 | -0.94286 | 0.016667* |
| 3 | Protein kinase byr2 | L484_007594.t01 | -0.94286 | 0.016667* |
| 4 | Detoxfication protein 19 | L484_009604.t01 | -0.98561 | 0.000309** |
| 5 | Protein farnesyltransferase subunit beta | L484_020495.t01 | -0.94286 | 0.016667* |
| **Perphenazine related genes** | | | | |
| 1 | Acyl-[acyl-carrier-protein] desaturase 6 | L484_003523.t01 | 0.942857 | 0.016667* |
| 2 | Desiccation-related protein PCC13-62 | L484_005955.t01 | -1 | 0.002778** |
| 3 | Detoxfication protein 19 | L484_009604.t01 | -0.92763 | 0.007666** |
| 4 | Hypothetical protein | L484_022566.t01 | -0.94286 | 0.016667* |
| 5 | Putative transporter MCH1 | L484_022567.t01 | -0.94286 | 0.016667* |
| **Pinoresinol diglucoside related genes** | | | | |
| 1 | Detoxfication protein 19 | L484_009604.t01 | 0.927634 | 0.007666** |
| 2 | Xylose isomerase | L484_022871.t01 | 0.942857 | 0.016667* |
| 3 | Topless-related protein 4 | L484_025769.t01 | -0.94286 | 0.016667* |
| **Quercetin 3-O-rhamnoside-7-O-glucoside related genes** | | | | |
| 1 | L-ascorbate peroxidase 3 | L484_024998.t01 | 0.942857 | 0.016667* |
| 2 | Topless-related protein 4 | L484_025769.t01 | -1 | 0.002778** |
| **Rutin related genes** | | | | |
| 1 | Flavonoid 3'-monooxygenase | L484_004711.t01 | 0.942857 | 0.016667* |
| 2 | Topless-related protein 4 | L484_025769.t01 | -0.94286 | 0.016667* |
| **Sinapic acid related genes** | | | | |
| 1 | ABC transporter C family member 8 | L484_004550.t01 | -0.94286 | 0.016667* |
| 2 | Desiccation-related protein PCC13-62 | L484_005955.t01 | -0.94286 | 0.016667* |
| 3 | Serine/threonine-protein kinase HT1 | L484_006079.t01 | 0.942857 | 0.016667* |
| 4 | DnaJ homolog subfamily B member 13 | L484_015060.t01 | -0.94286 | 0.016667* |
| 5 | Heat shock protein 83 | L484_024225.t01 | -0.94286 | 0.016667* |
| 6 | L-ascorbate peroxidase 3 | L484_024998.t01 | -0.94286 | 0.016667* |
| **Sinapinic acid related genes** | | | | |
| 1 | Acyl-[acyl-carrier-protein] desaturase 6 | L484_003523.t01 | 0.942857 | 0.016667* |
| 2 | Desiccation-related protein PCC13-62 | L484_005955.t01 | -1 | 0.002778** |
| 3 | Detoxfication protein 19 | L484_009604.t01 | -0.92763 | 0.007666** |
| 4 | Hypothetical protein | L484_022566.t01 | -0.94286 | 0.016667* |
| 5 | Putative transporter MCH1 | L484_022567.t01 | -0.94286 | 0.016667* |
| **Sudan III related genes** | | | | |
| 1 | Disease resistance-like protein | BGI_novel_G000537 | 1 | 0.002778** |
| 2 | Nicotinamidase 1 | L484_000190.t01 | 0.985611 | 0.000309** |
| **Vanillin related genes** | | | | |
| 1 | Morus notabilis protein NRT1 | BGI_novel_G000340 | 1 | 0.002778** |
| 2 | Nicotinamidase 1 | L484_000190.t01 | -0.92763 | 0.007666** |
| 3 | Morus notabilis cyclin-D3-1 | L484_027417.t01 | 1 | 0.002778** |
| **Xanthohumol related genes** | | | | |
| 1 | Cannabidiolic acid synthase | BGI_novel_G000544 | -0.94286 | 0.016667* |
| 2 | Putative receptor protein kinase TMK1 | L484_001220.t01 | -0.94286 | 0.016667* |
| 3 | Disease resistance protein RPM1 | L484_009582.t01 | -0.94286 | 0.016667* |
| 4 | Transcription factor bHLH130 | L484_010917.t01 | -1 | 0.002778** |
| 5 | Putative methyltransferase PMT18 | L484_011830.t01 | 0.941124 | 0.005098** |
| 6 | RNA-binding protein 24 | L484_012724.t01 | -0.94286 | 0.016667* |
| 7 | Inositol-3-phosphate synthase | L484_016608.t01 | 1 | 0.002778** |
| 8 | NADP-dependent alkenal double bond reductase P2 | L484_018505.t01 | 1 | 0.002778** |
| **β-Asarone related genes** | | | | |
| 1 | Homogentisate solanesyltransferase | BGI_novel_G001371 | -0.94286 | 0.016667* |
| 2 | Flavonoid 3'-monooxygenase | L484_004711.t01 | 1 | 0.002778** |
| 3 | Putative methyltransferase PMT18 | L484_011830.t01 | 0.941124 | 0.005098** |
| 4 | L-ascorbate peroxidase 3 | L484_024998.t01 | 0.942857 | 0.016667* |
